# A cross-sectional study of COVID-19-related bullying in a sample of Lebanese adults: scale validation, correlates, and mediating effect of fear and anxiety

**DOI:** 10.1186/s40359-021-00643-1

**Published:** 2021-09-08

**Authors:** Chadia Haddad, Hala Sacre, Sandrella Bou Malhab, Diana Malaeb, Danielle Saadeh, Christine Abou Tayeh, Pascale Salameh

**Affiliations:** 1 Research Department, Psychiatric Hospital of the Cross, P.O. Box 60096, Jal Eddib, Lebanon; 2 INSERM, CH Esquirol Limoges, IRD, U1094 Tropical Neuroepidemiology, Institute of Epidemiology and Tropical Neurology, GEIST, University of Limoges, Limoges, France; 3 INSPECT-LB (Institut National de Santé Publique, d’Épidémiologie Clinique et de Toxicologie-Liban), Beirut, Lebanon; 4 School of Pharmacy, Lebanese International University, Beirut, Lebanon; 5 Faculty of Public Health, Lebanese University, Beirut, Lebanon; 6 Faculty of Pharmacy, Lebanese University, Beirut, Lebanon; 7 University of Nicosia Medical School, Nicosia, Cyprus

**Keywords:** COVID-19, Bullying, COVID-19 Bullying Scale, Fear, Anxiety, Mediation, Scale validation

## Abstract

**Background:**

The COVID-19 pandemic has led to prolonged exposure to stress and anxiety, raising concerns about a large spectrum of psychological side effects. The primary objective of the study was to validate the COVID-19 Bullying Scale (CBS-11). The second objective was to explore factors associated with COVID-19-related bullying and evaluate the mediating effect of fear and anxiety between knowledge and COVID-19-related bullying.

**Methods:**

A cross-sectional online survey conducted between December 20, 2020, and January 5, 2021, recruited 405 Lebanese adults using a snowball sampling technique. The CBS-11, an 11-item tool specifically created for this study, was used to measure bullying behaviors towards COVID-19 patients.

**Results:**

All items of the CBS-11 converged over a 1-factor solution with an eigenvalue over 1, accounting for a variance of 75.16%. The scale has a high Cronbach’s alpha (.974), indicating excellent reliability. A positive correlation was found between the COVID-19 bullying scale and fear, anxiety, and stigma discrimination. The logistic regression showed that higher fear of COVID-19 (ORa = 1.04), a positive attitude toward COVID-19 preventive measures and hygiene recommendations (ORa = 1.18), higher stigma discrimination scores (ORa = 1.09), and having a health professional family member (ORa = 2.42) were significantly associated with bullying.

**Conclusion:**

Our main findings showed that the CBS-11 could be an efficient tool to measure bullying behaviors toward COVID-19 patients. Stigma discrimination and fear from COVID-19 were associated with higher bullying attitudes. Future prospective studies are needed to understand better the factors related to bullying among adults during a pandemic, such as COVID-19.

**Supplementary Information:**

The online version contains supplementary material available at 10.1186/s40359-021-00643-1.

## Background

The Coronavirus disease 2019 (COVID-19), caused by the Severe Acute Respiratory Syndrome Coronavirus 2 (SARS-CoV-2), is one of the century's main challenges. It was declared a pandemic a few months after the first detected case in Wuhan, China (on 17 November 2019). It spreads rapidly, affecting millions of people worldwide, with a mortality rate of 2.2%, as reported by the World Health Organization (WHO) in February 2021. The COVID-19 outbreak was sudden, unexpected, and unpredictable in most countries due to the heterogeneous healthcare systems [1]. The rapid spread of COVID-19 has raised global concerns as, in the absence of effective treatment, the remedies from the virus remain empirical and symptomatic [2–5]. Thus, to minimize the virus spread, efforts are mainly focusing on preventive measures, including social distancing, awareness through public health education, and hygiene practices in daily routines, in addition to sanitary lockdown [6]. In Lebanon, the first case of COVID-19 was detected in February 2020 [7], mandating several stepwise restrictive measures. With the sharply growing numbers, the government had imposed a total lockdown on public places, schools, and social settings to mitigate disease spread [7].

Lockdowns due to COVID-19 have unfavorably impacted lives in the community and at home, resulting in increased fear and uncertainty, which, in turn, could lead to negative societal behaviors [8], with an expected increase in bullying behaviors [9], stress, and anxiety, raising concerns regarding a large spectrum of psychological side effects [10]. Bullying is a form of abusive behavior defined as interpersonal aggression characterized by repeated intent to harm and control weaker people [11], with a global prevalence of 36%, varying considerably between countries [12]. It may be direct or indirect and may be verbal, physical, psychosocial, emotional, or virtual (cyberbullying) [13]. Bullying is a deliberate, aggressive behavior, repeated or not over time, involving an actual or perceived power imbalance, where power could be used to manipulate or damage others physically (hitting and kicking others) or verbally (threats, spreading rumors, verbally assaulting others, and purposefully removing someone from a group) [14].

Bullying and rejection could result from physical distancing that increase social isolation, separation from peers, and fear of contagion [15]. Distress is also common in COVID-19 patients due to the fear of losing family members, income, and employment [16]. Several factors are known to influence stigmatization and bullying towards COVID-19. People with higher personal resources (education, income) and good mental health (lower anxiety and fear) have better awareness regarding emergent infectious diseases and are less prone to discriminate and stigmatize [17, 18]. Thus, uncomplicated and transparent communication improves knowledge, attitude, and behavior towards COVID-19 and reduces fears, social stigma, and discrimination [19], referred to as treating a person unfairly or differently.

Insufficient knowledge and contradictory information about this emergent pandemic disease related to obligatory protective measures, such as wearing face masks in public, are associated with increased anxiety. Anxiety, partially due to lockdowns and fear of being infected, triggers stigmatization, discrimination, and attacks against vulnerable people suspected or diagnosed with COVID-19 in local communities [20]. According to the WHO, social stigma is a negative association between a person or a group of people who share certain characteristics and a specific disease [21]. In an outbreak, as in COVID-19, it may mean people are discredited, labeled, stereotyped, discriminated against, and reduced to a perceived link with disease [22]. Moreover, fear of community outbreaks and rumors spread on social media can result in growing anxiety and social panic [23, 24]. Thus, anxiety and fear from infected persons with COVID-19 can cause the community to blame and even compare patients to criminals. Infected people may then become a target of discrimination (which increased with the number of reported confirmed cases [25]), a source to be blamed for, and prone to stigmatization [1].

In Arab countries, where factors related to aggressive activity among children are mainly due to socio-political instability, bullying is prevalent [26]. In Lebanon, higher exposure to war events was correlated with a greater likelihood to experience other acts of interpersonal violence [27]. A study among 510 Lebanese adolescents revealed that around 30% of participants were involved in bullying [28], while another among 1810 Lebanese adolescents showed that 46.5% were previously bullied [29].

Most studies about bullying cover adolescents and working life exclusively, investigating bullying prevalence and incidence mainly [12, 30–32]. Validation studies about bullying are limited and still under considerable debate due to the different instruments and approaches used to measure various bullying types [33]. The most commonly used scale is the Forms of Bullying Scale (FBS), a multi-item, valid, and reliable tool used to assess different forms of bullying victimization and perpetration [34]. The only available scale for adults in the literature was the Bullying Scale for Adults (BSA), consisting of 21 items used to measure bullying experiences and activities [35]. The BSA has very good internal consistency (Cronbach’s alpha = .93) and good concurrent validity to assess various aspects of bullying among adults with different mental disorders [35]. The different scales available in the literature did not serve the purposes of our study. For example, they measure specific aspects among specific populations (such as victimization [36, 37] or victimization and perpetration among adolescents [34]), and some are long with a large number of items [37]; the only scale for adults (BSA) was used among psychiatric patients [35].

To our knowledge, no research has yet assessed bullying towards patients infected with COVID-19 in the general population, except for one study conducted among 7411 healthcare workers (HCWs) from 173 countries, which used one question to evaluate bullying: “Have you or a family member been harassed, bullied, or hurt because of coronavirus?” and this showed that HCWs are significantly more likely to experience COVID-19-related bullying [38]. The lack of tools that assess bullying in the general adult population has led to developing a bullying assessment scale. Therefore, the primary objective of the study was to validate the COVID-19 Bullying Scale (CBS-11). The second objective was to explore factors associated with COVID-19-related bullying and evaluate the mediating effect of fear and anxiety between knowledge and COVID-19-related bullying.

## Methods

### Study design and sampling

A cross-sectional online survey conducted between December 20, 2020, and January 5, 2021, involved 405 Lebanese adults recruited using a snowball sampling technique. An anonymous self-administered questionnaire was developed on Google Forms and posted on different social media sites (WhatsApp, Facebook, and Instagram). Participants were asked to share the questionnaire with their peers and contacts. All people above the age of 18 who had access to the internet were eligible to participate in the study. Participants were aware of the purpose of the study and gave informed consent before enrolling. They received no compensation in return for their participation, which was entirely voluntary.

### Sample size calculation

The G-power software calculated a minimum sample of 311 participants based on an effect size *f* = 2%, an alpha error of 5%, a power of 80%, and considering ten factors to be entered in the multivariable analysis.

### Questionnaire

The online survey tool (Additional file 1), which included open and closed-ended questions, was available in English and Arabic and consisted of two sections.

The first part of the questionnaire consisted of sociodemographic characteristics such as age, gender, marital status, educational level, monthly income, employment status, having a family member working in the medical field, the region of residence, the number of people living in the house, the number of rooms in the house, and religion. It also included questions related to having been diagnosed with COVID-19, having ever tested for COVID-19, and having a family history of COVID-19. The household crowding index was calculated by dividing the number of persons living in the house by the number of rooms, excluding the kitchen and bathrooms. The monthly income was divided into four levels: no income, low < 1,500,000 LBP (Lebanese Pounds), intermediate 1,500,000–2,000,000 LBP, and high income > 2,000,000 LBP.

The second part of the questionnaire included the scales used in this study:

### COVID-19 Bullying Scale (CBS-11)

The COVID-19 Bullying Scale is an 11-item tool specifically created for this study to measure the behaviors of people engaging in bullying towards COVID-19 patients. Due to the scarcity of bullying scales for adults, in general, and in the absence of a tool assessing bullying towards COVID-19 patients, the CBS-11 was inspired by four previous studies: three evaluated bullying behaviors and cyberbullying among adolescents [34, 39, 40], and one assessed bullying among adults [34]. The scale included 12 questions initially, but one was removed based on the factor analysis results. All eleven items were scored on a 5-point Likert scale: 1 = never, 2 = once or twice, 3 = a few times (between 3 and 5), 4 = several times (between 6 and 10), and 5 = many times (more than 10). Examples of the items include: “I teased someone in my surrounding when I knew they tested COVID-19 positive,” “I hurt someone with COVID-19/or suspected having COVID-19 by trying to break up a friendship,” “I have told lies and/or spread rumors about someone with COVID-19 to make their friends or others not talk to him.” The total bullying score was created by summing the eleven questions ranged from 11 to 55, with higher scores indicating higher bullying toward COVID-19 patients. In this study, Cronbach’s alpha was .974.

### The stigma discrimination scale (SDS-11)

At the time of the study, no tool was available to evaluate COVID-19-related stigma among the general population, except for one related to COVID-19 self-stigma among healthcare workers after quarantine in Vietnam [41]. After a rigorous literature review [42–46], an 11-item scale was developed and adapted to the Lebanese context to measure stigma discrimination during COVID-19, using questions selected from previous studies [42–44]. All items are rated on a 5-point Likert scale from 1 (strongly disagree) to 5 (strongly agree). Examples of the items include: “You feel it is not worthwhile for you to serve persons who contracted COVID-19,” “People with current COVID-19 are dangerous to the society,” “People with current COVID-19 should not have the same freedoms as other people.” The total stigma discrimination score calculated by summing all the answers ranges from 11 to 55, with higher scores indicating higher stigma discrimination. In this study, Cronbach’s alpha was .917.

### Fear of COVID-19 scale (FCV-19S)

The FCV-19S is a 7-item scale designed to measure the fear of COVID-19 in the general population [47]. In this study, the Arabic validated version of the FCV-19S was used [48]. The items are rated on a 5-point Likert scale ranging from 1 (strongly disagree) to 5 (strongly agree). Examples of the items include: “I am most afraid of coronavirus-19,” “My hands become clammy when I think about coronavirus-19,” “I cannot sleep because I’m worrying about getting coronavirus-19.” The total score was determined by adding the scores of each item. A higher score indicates a greater fear of COVID-19 [47]. In this study, Cronbach’s alpha was .856.

### Coronavirus anxiety scale (CAS)

The CAS is a 5-item self-report scale that measures physiologically-based symptoms caused by COVID-19-related facts and thoughts [49]. Participants are asked to rate how often they experienced each symptom of anxiety. The measure is rated on a 5-point Likert scale ranging from 0 (not at all) to 4 (extremely). Examples of the items include: “I felt dizzy, lightheaded, or faint, when I read or listened to news about the coronavirus,” “I felt paralyzed or frozen when I thought about or was exposed to information about the coronavirus,” “I felt nauseous or had stomach problems when I thought about or was exposed to information about the coronavirus.” The overall score is determined by adding the five items together, with higher scores reflecting higher anxiety about COVID-19 [49]. In this study, Cronbach’s alpha was .846.

### Knowledge, attitude, and practice (KAP) toward the COVID-19 pandemic

The questions used to evaluate KAP toward COVID-19 were adapted from previous studies conducted among health practitioners and the general population [50–56]:

*Knowledge scale*: twenty multiple-choice items (with single- and multiple-option answers) assessed the knowledge regarding the COVID-19 disease. All answers were coded as binary variables (1 = Yes, 0 = No); multiple-option questions were considered separate variables. Examples of the items include: “For how long should a person be isolated in case of COVID-19 infection suspicion (mild symptoms or contact with an infected persons),” “If a suspected person tests negative but has no symptoms,” Can someone who has been quarantined for COVID-19 spread the illness to others?” The total score calculated by summing all the correct answers ranged from 0 to 29, where higher scores indicate higher knowledge about COVID-19. In this study, Cronbach’s alpha was .557.

*Attitude scale*: six questions assessed attitudes toward COVID-19 preventive measures and hygiene recommendations. All items are graded on a 3-point Likert scale from 1 (disagree) to 3 (agree). Examples of the items include: “Do you think social distancing/self-isolation is an effective measure to reduce the spread of COVID-19?” “Keeping up with the information regarding the government’s call for COVID-19 preventive efforts is important for the community,” “People with COVID-19 who isolate themselves show that they have a responsibility in preventing the transmission of COVID-19”. The overall attitude score calculated by adding the six responses ranged from 6 to 18, with a higher score indicating a more positive attitude toward COVID-19. In this study, Cronbach’s alpha was .615.

*Practice scale*: twenty-four questions evaluated practices and behaviors regarding preventive measures against COVID-19, from 1 (never, the worst) to 5 (always, the best). Examples of the items include: “In the last few days, have you worn a mask when you were in a crowded place?” “Do you maintain social distance (or home quarantine)?” “Do you clean/disinfect mobile phone?” The total practice score calculated by summing the 24 items ranged from 24 to 120, with higher scores indicating appropriate safety practice towards COVID-19. In this study, Cronbach’s alpha was .900.

### Translation and adaptation procedure

The COVID-19 Bullying Scale and the other scales used were translated from English to Arabic using the forward and backward translation process, except for the FCV-19S. Two authors performed the translation from English to Arabic, and another two authors did the back-translation. Discrepancies between the original English version and the translated edition were resolved by consensus. All the authors reviewed and approved the translation and contributed to cultural adaptations.

### Pre-testing

A pilot study was conducted on ten subjects to check the clarity of the questionnaire and test for the acceptability of questions. Related data were included in the final dataset and they did not affect neither negatively or positively on the current study result.

### Statistical analysis

Data were analyzed on SPSS software version 25. A descriptive analysis was performed using absolute frequencies and percentages for categorical variables and means and standard deviations (SD) for quantitative measures.

Construct validity of the Bullying Scale was assessed using the principal component analysis. Kaiser–Meyer–Olkin’s measure of sampling adequacy and Bartlett’s test of sphericity were calculated to ensure the model’s adequacy. Factors with eigenvalues values greater than one were retained, and the scree plot method was used to determine the number of components to extract [57]. Only items with factor loading greater than 0.4 were considered [58]. Moreover, the internal consistency of the COVID-19 Bullying Scale was assessed using Cronbach's alpha. Spearman correlation analysis was used to estimate the relationships of bullying items and the whole scale scores and with fear, anxiety, and Stigma Discrimination Scale toward COVID-19.

Since the Bullying Scale was not normally distributed, it was dichotomized into two groups: the presence and absence of bullying. The Chi-square and Fisher exact tests were used to compare categorical variables and the Mann–Whitney test to compare continuous variables. Bullying was considered if the score was over zero, while a score of 0 indicated the absence of bullying. A backward logistic regression was conducted, taking the variable presence/absence of bullying as the dependent variable. All the variables that showed a *p*-value < .2 in the bivariate analysis were included in the model to eliminate potential confounding factors.

A mediation analysis was run to measure the mediating effect of fear and anxiety on the Bullying Scale as the dependent variable and the knowledge scale as the independent variable. Pathway A determined the regression coefficient for the effect of knowledge on fear and anxiety. Pathway B examined the association between anxiety and fear with bullying, independent of knowledge level, and Pathway C estimated the total and direct effect of knowledge on bullying. Pathway AB calculated the indirect intervention effects. The Sobel test was performed to calculate the significance of the mediation effect [59]. The covariates that were included in the mediation model were those that showed significant associations with the bullying scale in the bivariate analysis. A *p*-value less than .05 was considered significant.

## Results

### Sociodemographic characteristics of the studied sample

The mean age of the participants was 28.38 ± 12.02 years, and the mean household crowding index was 1.14 ± 0.55. The majority of the participants were females (79.8%), single (68.1%), living in an urban area (73.3%), Muslim (62.7%), unemployed (55.8%), with a university education (89.6%). Only 10.1% of the participants had been diagnosed with COVID-19, 40.5% had tested for COVID-19, and 27.9% had someone in their family who contracted COVID-19. The sociodemographic characteristics of the participants are summarized in Additional file 2.

### Description of the scales used in the study

Table 1 describes the scales used in this study. The mean bullying score was 14.2 ± 5.6, and the median was 13. In the absence of a cutoff score for this scale, the total score was categorized into two groups according to the presence and absence of bullying; the results showed that 240 (59.3%, CI .46–.51) participants bullied other people for having COVID-19.

**Table 1:** Descriptive statistics for the study measures

	Mean	SD	Median	Minimum	Maximum
COVID-19 Bullying scale	12.4	5.4	11.0	11.0	55.0
Stigma Discrimination Scale	33.4	4.8	33.0	19.0	55.0
Fear of COVID-19 scale	17.5	5.5	17.0	7.0	32.0
Anxiety from COVID-19 scale	1.2	2.4	1.0	0.0	16.0
Knowledge of COVID-19 scale	20.4	3.5	21.0	5.0	27.0
Positive attitude toward COVID-19 preventive measures and hygiene recommendations	16.5	1.8	17.0	6.0	18.0
Positive practice toward preventive measures against COVID-19	104.6	12.5	109.0	50.0	120.0

### Factor analysis of the COVID-19 bullying scale

All items of the Bullying Scale could be extracted from the list, and the scale converged over a 1-factor solution with an eigenvalue over 1, accounting for a variance of 75.16% (Bartlett sphericity test *P* < .001, KMO = .909). The components of the scale are presented in Table 2.

**Table 2:** Promax rotated matrix of bullying

Factor	Item	Factor 1	H2 communalities
I hurt someone with COVID-19/ or suspected having COVID-19 by trying to break up a friendship	3	.969	.940
I have told lies and/or spread rumors about someone with COVID-19 to make their friends or others not talk to him	7	.967	.935
I have ignored someone with COVID-19 and did not answer messages or things he shared in groups or social networks, just to make him feel bad	10	.962	.924
I have sent or posted mean or hurtful pictures/videos on social networks to someone with COVID-19	9	.951	.905
I have insulted or ridiculed someone with COVID-19 on social networks or groups like WhatsApp and Facebook to really annoy him	8	.947	.896
I have called someone having COVID-19 with hurtful names	2	.946	.895
I have eliminated or blocked someone with COVID-19 from groups to leave him/her without any friends	11	.933	.870
I teased someone in my surrounding when I knew he tested COVID-19 positive	1	.892	.796
I have refused to talk with someone having COVID-19	5	.835	.698
I have ignored someone having COVID-19	4	.818	.669
I would not let someone having COVID-19 to join my friendship group	6	.683	.466
Percentage variance explained		75.165%	
Chronbach alpha = .974			
Kaiser–Meyer–Olkin (KMO) = .909			
Bartlett’s test of sphericity *p* < .001			

### Intercorrelations of the COVID-19 bullying scale (CBS-11) items

Table 3 presents the correlations between the items of CBS-11. All correlations between items and the total bullying score were significant (*p* < .05).

**Table 3:** Intercorrelations of the bullying toward covid-19 scale items

	Total scale	Item 1	Item 2	Item 3	Item 4	Item 5	Item 6	Item 7	Item 8	Item 9	Item 10
Item 1	.420**										
Item 2	.342**	.719**									
Item 3	.386**	.836**	.862**								
Item 4	.485**	.485**	.516**	.532**							
Item 5	.528**	.615**	.553**	.644**	.661**						
Item 6	.507**	.479**	.482**	.532**	.473**	.552**					
Item 7	.376**	.815**	.885**	.974**	.514**	.629**	.517**				
Item 8	.358**	.743**	.854**	.889**	.532**	572**	.499**	.913**			
Item 9	.379**	.746**	.863**	.894**	.499**	.575**	.464**	.917**	.830**		
Item 10	.372**	.767**	.887**	.918**	.514**	.589**	.519**	.943**	.914**	.915**	
Item 11	.362**	.743**	.857**	.889**	.534**	.572**	.499**	.913**	.942**	.888**	.971**

Significant correlation at: *p < .05; **p < .001

A positive correlation was found between COVID-19 bullying scale and fear, anxiety, and stigma discrimination scale (Table 4).

**Table 4:** Correlation between the scales used in the study and the COVID-19 bullying scale

	COVID-19 Bullying Scale	*p*-value
Fear of COVID-19	.129	.009
Anxiety of COVID-19	.127	.011
Stigma Discrimination Scale	.317	< .001

### Bivariate analysis

The bivariate analysis taking the presence/absence of bullying as the dependent variable showed that higher bullying behaviors were associated with males, married participants, higher fear of COVID-19, higher stigma discrimination, having a family member working in the medical field, and positive attitudes toward COVID-19 preventive measures and hygiene recommendations (Table 5).

**Table 5:** Bivariate analysis of the categorized bullying COVID-19 scale as the dependent variable

	COVID-19 Bullying Scale		*p*-value
	Absence of bullying (n = 165 (40.7%))	Presence of bullying (n = 240 (59.3%))	
*Gender*			
Male	22 (26.8%)	60 (73.2%)	**.004**
Female	143 (44.3%)	180 (55.7%)	
*Marital status*			
Single	124 (44.9%)	152 (55.1%)	**.012**
Married	41 (31.8%)	88 (68.2%)	
*Having a family member in the medical field*			
Yes	29 (25.4%)	85 (74.6%)	**< .001**
No	136 (46.7%)	155 (53.3%)	
*History of COVID-19 in the family*			
Yes	62 (54.9%)	51 (45.1%)	**< .001**
No	103 (35.3%)	189 (64.7%)	
	Mean ± SD	Mean ± SD	
Age	27.27 ± 10.02	29.12 ± 13.18	.110
Household crowding index	1.09 ± 0.52	1.17 ± 0.57	.118
Fear of COVID-19	16.80 ± 5.99	17.94 ± 5.16	**.047**
Anxiety of COVID-19	1.09 ± 2.19	1.22 ± 2.61	.588
Stigma discrimination scale	24.52 ± 5.38	27.44 ± 5.12	**< .001**
Knowledge toward COVID-19	20.32 ± 3.20	20.43 ± 3.71	.757
Attitude of COVID-19	16.18 ± 1.79	16.74 ± 1.79	**.002**
Practice of COVID-19	103.55 ± 12.26	105.40 ± 12.72	.145

Note: Values marked in bold are significant

### Multivariable analysis

A logistic regression, taking the presence/absence of bullying as the dependent variable, showed that higher fear of COVID-19 (ORa = 1.04), positive attitudes toward COVID-19 preventive measures and hygiene recommendations (ORa = 1.18), higher stigma discrimination (ORa = 1.09), and having a family member in the medical field (ORa = 2.42) were significantly associated with the presence of bullying. Being a female (ORa = .48) and having someone in the family who contracted COVID-19 (ORa = .56) were significantly associated with the absence of bullying (Table 6).

**Table 6:** Multivariable analysis

Model 1: Logistic regression taking the categorized bullying scale (presence/absence) as the dependent variable				
	*p*-value	ORa	Confidence interval	
			Lower bound	Upper bound
Gender (female vs. male*)	.020	.487	.265	.893
Someone in the family contracted COVID-19	.024	.566	.345	.928
Fear of COVID-19	.053	1.040	1.000	1.083
Attitude scale	.008	1.180	1.045	1.332
Stigma discrimination scale	< .001	1.094	1.048	1.142
Having a family member in the medical field	.001	2.422	1.434	4.092

Variables entered gender, marital status, history of COVID-19 in the family, fear of COVID-19 and positive attitude toward COVID-19 scale

^*^Reference group

### Mediation analysis

Table 7 describes the mediation analysis conducted on the knowledge score. The fear score had no mediating effect on the association between the knowledge scale and bullying; however, the anxiety scale significantly mediated the association between the knowledge score and the bullying scale.

**Table 7:** Mediating effects of fear and anxiety scores

Model 1: Linear regression taking the fear score as dependent variable and knowledge score as independent variable					
Factor	UB (SD)	SB	95% CI	*p*-value	*p*-value (mediating factor)
Knowledge score	.071 (.079)	.045	− .083; .226	.365	
Model 2: Linear regression taking the bullying score as dependent variable and knowledge and fear scores as independent variables					
Factor	UB (SD)	SB	95% CI	*p*-value	
Knowledge score	− .176 (.079)	− .110	− .332; − .021	.026	
Fear score	.131 (.050)	.120	.032; .229	.010	.395
Model 3: Linear regression taking the anxiety score as dependent variable and knowledge score as independent variable					
Factor	UB (SD)	SB	95% CI	*p*-value	*p*-value (mediating factor)
Knowledge score	− .115 (.034)	− .165	− .182; − .048	.001	
Model 4: Linear regression taking the bullying score as dependent variable and knowledge and anxiety scores as independent variables					
Factor	UB (SD)	SB	95% CI	*p*-value	
Knowledge score	− .071 (.075)	− .044	− .219; .077	.347	
Anxiety score	.835 (.108)	.362	.622; 1.048	< .001	.002
Model 5: Linear regression taking the bullying score as dependent variable and knowledge score as independent variable					
	UB (SD)	SB	95% CI	*p*-value	
Knowledge score	− .167 (.08)	− .104	− .324; − .010	.037	

*UB* unstandardized beta, *SB* standardized beta, *SD* standard deviation, *CI* confidence interval

## Discussion

Based on previous papers [34, 39, 40], this study could create and validate the COVID-19 Bullying Scale (CBS-11), an 11-item easy-to-understand tool used to measure bullying towards COVID-19 patients in adults. It also evaluated the rate of the bullying act and assessed the related factors among a sample of Lebanese adults.

The CBS-11 showed to be efficient, with an average completion time of five minutes. Its internal consistency was high in our sample, indicating that the scale is reliable. Also, the convergent validity with other used scales was appropriate, and the correlation of the items with CBS-11 displayed favorable results. The factor analysis of CBS-11 showed that all items had high loadings on one factor, reflecting a robust factorial validity. These results indicate that CBS-11 is a valid and reliable tool to assess bullying toward COVID-19 patients among the general adult population, which could help in research and clinical practice. However, our findings were not comparable to those in the literature since no scale is available to assess bullying among adults during infectious disease outbreak, and the CBS-11 was created especially for this study; hence the need for further studies to confirm our results.

Bullying is a global public health problem mainly prevalent among students and labor forces [60], expected to increase during lockdowns due to the increased tensions between individuals [61]. In our study, 59.3% of participants reported having been bullied during this pandemic, a high proportion that is not comparable to any other results as, to date, no research has yet evaluated bullying toward COVID-19 patients in the general adult population. Nevertheless, a study among 7411 healthcare from 173 countries found that 8.0% of the participants endured COVID-19-related harassment, bullying, or hurt [38]. Other available studies are related to bullying among adolescents, where the prevalence ranges from 8 to 55% [29, 62–65]. The high bullying rate in our study could be due to the type of scale used to measure bullying, different from those previously used among adolescents [34, 40]. Also, contextual characteristics of surrounding communities could have influenced bullying behaviors and the perception of people with an infectious disease [66, 67]. For example, the family could be considered a protective factor and might play a role in bullying prevention [68]. Lebanon and other Arab countries share common cultural values such as power gap, ambiguity avoidance, and masculinity [69]. Some Arab families might protect their members from bullying, while others might reinforce behaviors that expose their children to bullying [26].

Our results revealed that those who have discrimination attitudes exhibit more bullying behaviors toward COVID-19 patients. It is well known that bullying and discrimination have several similar and overlapping characteristics [70]. Both can happen once or repeatedly over time. Both depend on the imbalances between the person who engages in the bullying act and the target [70]. Epidemic outbreaks such as HIV/AIDS, SARS, Ebola virus disease, and Zika virus were historically associated with discrimination and negative attitudes toward the infected persons [71]. A study conducted in an urban Southern Africa setting showed an association between stigma, bullying, and mental health in people with HIV [72]. Another clinical-based study of HIV-positive adolescents found that HIV-related stigma and bullying were associated with major depressive disorder and a high risk of suicidality [73].

In our study, a higher fear of COVID-19 was significantly associated with the act of bullying, likely due to increased coronavirus-induced anxiety, resulting from news updates about complications and death reports from COVID-19 [74]. Fear of COVID-19 substantially increases negative feelings such as anxiety and depression [75]. In response to this fear of disease and death, people tend to blame others; they also engage in gossips and spread rumors and misconceptions [75]. An online study of 3551 non-healthcare workers in the US and Canada revealed that a high percentage of Canadians and Americans believed that healthcare workers should not be allowed to go out in public, should have restrictions on their freedoms, should be isolated from the community, and separated from their families [76].

In our study, participants having someone in their family who contracted COVID-19 were less likely to engage in bullying acts, contrary to previous findings showing that US adults who experienced COVID-19 (e.g., being diagnosed with COVID-19 or knowing people with COVID-19) were more likely to engage in cyberbullying behaviors [77]. Moreover, an Iranian study among 1498 participants found that having a family member with COVID-19 was associated with higher anxiety levels [78]. Our results support the idea that the family might be supportive and might play a protective role against bullying, particularly after acquiring sufficient knowledge about the disease, thus reducing unfavorable psychological effects due to COVID-19. Social support helps individuals to cope with the detrimental effects of the disease by mitigating anxiety and fear. A systematic review of meta-analyses unveiled the most important protective factors against bullying and cyberbullying in the community, school, family, and peers, showing that having a family member in the medical field increases bullying acts [79]. Similarly, a study has reported that healthcare workers are more subject to harassment and bullying [38, 79].

Our results showed that adherence to prevention strategies and recommendations was related to higher bullying, contrary to previous findings showing that the fewer healthcare workers adhere to the prevention measures, the more exposed they were to COVID-19 bullying [38]. One explanation for our results could be that people wearing masks and adhering to the protective measures in public and crowded places are subject to bullying and intimidation by those who deny coronavirus and are opposed to hygiene measures. Further studies are needed to elucidate these discrepancies.

Anxiety due to COVID-19 mediated the association between knowledge and COVID-19 bullying scale in our study. No similar framework exploring the relationships between these factors is available in the literature. Most studies investigate psychological pathways between adolescents and bullying [29, 80, 81]. Psychological distress can result from a lack of awareness and misconceptions regarding COVID-19 [82–84]. Further studies are needed to identify factors that promote and mediate the bullying process of individuals.

Moreover, our study showed that women were less likely to engage in bullying acts than men. Bullying others has been described as a gendered phenomenon [85]. In England, more than one-quarter of males (26.9%) and one-in-seven females (14.8%) reported that they had bullied others [86]. A study in Taiwan evaluating the prevalence of school bullying among secondary students showed higher bullying acts rates in male students compared to female students [87]. Every society categorizing people according to sex, assigns specific expectations that are part of the gender social construction [88]. Lebanon is described as a patriarchal country, where men have the authority and power, thus explaining our results [89]. Also, it could be that both genders exhibit bullying but under different forms; boys are more likely to express physical bullying behaviors such as hitting and fighting while girls usually engage in indirect bullying such as teasing, gossiping, or spreading rumors [90].

### Strengths and limitations

This study was the first to provide an insight into bullying among a sample of adults from the general population. It can serve to create new research questions or hypotheses about drivers for bullying and related factors. However, it has several limitations. Its cross-sectional design does not allow us to infer causality between bullying and the associated variables, essentially fear, anxiety, and stigma toward COVID-19. The fact that data were gathered online using a self-reported questionnaire could have generated an information bias. However, to reduce this kind of bias, we have used odd-numbered response categories with a neutral option, allowing the participants to opt-out and give an indifferent response, resulting in a non-differential measurement error instead of a differential information bias. The link to the survey was only available for a few days; it is therefore advisable to continue collecting data to explore changes in mental health over time. The sample size was not big enough to generalize to the whole population. The snowball technique used to collect the data is a nonrandom technique that could generate a selection bias. The bullying scale was created especially for this study and was not yet validated. Residual confounding bias is also possible since some bullying-related factors such as personality traits, aggressiveness, and family support, were not assessed in this study. Lastly, there are very few studies on bullying in adults, limiting our ability to compare our results with those in the literature.

## Conclusion

Our main findings show that the COVID-19 Bullying Scale could be an efficient tool to measure bullying behaviors toward COVID-19 patients in the Lebanese adult population. Stigma discrimination and fear from COVID-19 were associated with higher bullying attitudes. Thus, public health strategies should promote credible and reliable information about the disease to decrease stress and fear in the community. Future prospective studies are needed to understand better the factors related to bullying among adults during a pandemic, such as COVID-19.

## Supplementary Information

**Additional files 1, 2**. Bullying and COVID-19 questionnaire. **Table S1**: Sociodemographic characteristics of the study sample.

## Data Availability

The datasets used and/or analyzed during the current study are available from the corresponding author on reasonable request.

## Abbreviations

COVID-19: Coronavirus disease 2019
SARS-CoV-2: Severe acute respiratory syndrome coronavirus 2
WHO: World Health Organization
FBS: Forms of Bullying Scale
BSA: Bullying Scale for Adults
CBS-11: COVID-19 Bullying Scale
LBP: Lebanese Pounds
SDS-11: Stigma Discrimination Scale
FCV-19S: Fear of COVID-19 scale
CAS: Coronavirus Anxiety Scale
KAP: Knowledge, attitude, and practice
SPSS: Statistical Package for the Social Sciences
SD: Standard deviation
CI: Confidence interval
KMO: Kaiser–Meyer–Olkin
ORa: Adjusted odds ratio
UB: Unstandardized beta
SB: Standardized beta
