# Supplementary material for: A cross-sectional study of COVID-19-related bullying in a sample of Lebanese adults: scale validation, correlates, and mediating effect of fear and anxiety
Source: BMC Psychol. 2021 Sep 8;9:137. doi: 10.1186/s40359-021-00643-1 (PMC8425023; doi:10.1186/s40359-021-00643-1)
Supplement: Supplementary file 1 — Additional files 1, 2. Bullying and COVID-19 questionnaire. Table S1: Sociodemographic characteristics of the study sample. [file 40359_2021_643_MOESM1_ESM.docx]

**Additional file 1**

**A Cross-Sectional Study of COVID-19-Related Bullying in a Sample of Lebanese Adults: Scale Validation, Correlates, and Mediating Effect of Fear and Anxiety**

**Chadia Haddad^1,2,3^, Hala Sacre**^3^**, Sandrella Bou Malhab^3^, Diana Malaeb^3,4^, Danielle Saadeh^3,5^, Christine Abou Tayeh^3^, Pascale Salameh^3,6,7^**

1. Research department, Psychiatric Hospital of the Cross, Jal Eddib, Lebanon
2. INSERM, Univ. Limoges, CH Esquirol Limoges, IRD, U1094 Tropical Neuroepidemiology, Institute of Epidemiology and Tropical Neurology, GEIST, Limoges, France
3. INSPECT-LB (Institut National de Santé Publique, d’Épidémiologie Clinique et de Toxicologie-Liban), Beirut, Lebanon
4. Doctor in clinical pharmacy, School of Pharmacy, Lebanese International University, Beirut, Lebanon
5. Faculty of Public Health, Lebanese University, Beirut, Lebanon
6. Faculty of Pharmacy, Lebanese University, Beirut, Lebanon
7. University of Nicosia Medical School, Nicosia, Cyprus

**Corresponding author:** Chadia Haddad. Psychiatric Hospital of the Cross, P.O. Box 60096, Jall-Eddib, Lebanon. Email address: [Chadia_9@hotmail.com](mailto:Chadia_9@hotmail.com)

**Additional file 1**

**Bullying and COVID-19 questionnaire**

As the COVID-19 pandemic progresses worldwide, the fear of infection increases and, with it, the stigma-discrimination of people, adding to the problem. This study is conducted by a group of academic researchers and focuses on the societal perception toward COVID-19. All the information gathered is anonymous and will be treated confidentially. Your participation in this study is voluntary.

Completing the questionnaire requires 10 to 15 minutes and indicates your consent to participate.

Thank you in advance for your time and participation.

مع تفاقم جائحة كورونا في جميع أنحاء العالم، يزداد الخوف من العدوى ومعه وصمة العار والتمييز ضد الناس، مما يزيد من المشكلة. هذه الدراسة تجريها مجموعة من الباحثين الأكاديميين وتركّز على النظرة المجتمعية تجاه كورونا. جميع المعلومات التي سيتم جمعها غير اسمية وسيتم التعامل معها بسرية تامة.

إن المشاركة في هذه الدراسة طوعية. إكمال الاستبيان يستغرق 10-15 دقيقة ويشير الى الموافقة على المشاركة.

شكراً سلفاً على وقتكم ومشاركتكم.

**Please check all the boxes to proceed to the survey/الرجاء اختيار جميع المربعات لبدء الاستبيان**

I have read and understood the above information sheet / أقر بأنني قرأت وفهمت ورقة المعلومات لمشروع البحث المذكور أعلاه

I understand that my participation is voluntary / أدرك أن مشاركتي اختيارية

I understand that my data will be kept confidential/ أعي أن المعلومات التي سأقدمها سيتم حفظها واستخدامها بسرية

I agree to take part in this study / أوافق على المشاركة في البحث المذكور أعلاه

**SECTION 1: DEMOGRAPHICS AND OTHER CHARACTERISTICS**

1. **Age_________________**

**العمر: ___________ سنة**

1. **Gender:** Male Female

الجنس: ذكر انثى

1. **Marital status:**

Married Single Widowed Divorced

**الوضع الاجتماعي:**

متزوج(ة) أعزب (عزباء) ارمل(ة) مطلق(ة)

1. **Education level**

Illiterate Primary Complementary Secondary University

**المستوى التعليمي**

أمي إبتدائي تكميلي ثانوي جامعي

1. **Monthly income**

No income Low (< 1.500.000 LL)

Intermediate (1.500.000-3.000.000 LL) High (>3.000.000 LL)

**الدخل الشهري**

لا دخل منخفض (أقل من 1.500.000 ل ل)

متوسط ​​(1.500.000 – 3.000.000 ل ل ) مرتفع (أكثر من 3.000.000 ل ل )

1. **Employment status**

Employed, Medical field (frontline contact with COVID-19 patients)

Employed, Medical field (non- frontline contact)

Employed, Non-medical

Unemployed

**الوضع الوظيفي**

موظف (ة) في المجال الطبي (يعمل في الخطوط الأمامية لمكافحة فيروس كوفيد -19)

موظف (ة) في المجال الطبي (لا يمعل في الخطوط الأمامية)

موظف (ة) , لا يعمل في المجال الطبي

عاطل عن العمل

1. **Do you have a family member who works in the medical field (first line contact with COVID-19 patients)?**

Yes No

**هل لديك أحد من أفراد الأسرة يعمل في المجال الطبي (الخطوط الأمامية لمكافحة مرض كوفيد -19)**

نعم لا

1. **Living place**

Rural Urban

**مكان الإقامة الحالي:**

الريف المدينة

1. **Area of residence**

Mount Lebanon Beirut North South Beqaa

Akkar Nabatieh Baalbeck / Hermel

**محافظة السكن الحالي**

جبل لبنان بيروت الشمال الجنوب البقاع عكار

نبطيه بعلبك / الهرمل

1. **Religion**

Christian Muslim Druze Atheist Refuse to answer

Other______

**الدين**

مسيحي مسلم درزي ملحد ارفض الإجابة

أخرى ______

1. **Number of persons living in the house, including you: ___________**

**ما هو عدد الأشخاص الذين يعيشون في المنزل بمن فيهم أنت: _________**

1. **Number of rooms in your house, excluding the kitchen and bathrooms____________**

**ما هو عدد الغرف في منزلك عدا المطبخ والحمامات: _____________**

1. **Is there a history of coronavirus in any of your family members?**

Yes No I do not know

**هل هناك إصابة بفيروس كورونا في أي من أفراد عائلتك؟**

نعم لا لا أعرف

1. **Have you been diagnosed with COVID-19?**

Yes No

**هل تم تشخيصك بكوفيد -19؟**

نعم لا

1. **Have you been tested for COVID-19?**

Yes No

**هل تم اختبارك من أجل كوفيد -19؟**

نعم لا

1. **Have you been quarantined (restriction on the movement)?**

Yes No

**هل تم وضعك في الحجر الصحي ؟**

نعم لا

1. **Have you had indirect contact (being in the same place, contact with the surfaces or objects used by the infected person) with someone diagnosed with COVID-19?**

Yes No I do not know

**هل كان لديك أي اتصال غير مباشر (تواجد في نفس المكان، ملامسة الأسطح أو الأشياء المستخدمة من الشخص المصاب) مع شخص تم تشخيص إصابته بكوفيد - 19**

نعم لا لا أعرف

1. **Have you had direct/close contact (less than 2 meters for 15 minutes) with someone who have COVID-19?**

Yes No I do not know

**هل كان لديك أي اتصال مباشر/وثيق مع شخص تم تشخيص إصابته بكوفيد - 19 (تواجد معه أقل من مترين لمدة 15 دقيقة)**

نعم لا لا أعرف

1. **Have you had direct/close contact with someone suspected of having COVID-19 (having respiratory symptoms)?**

Yes No I do not know

**هل كان لديك اتصال مباشر /وثيق مع شخص يشتبه في إصابته بكوفيد - 19 (يعاني من أعراض تنفسية)؟**

نعم لا لا أعرف

1. **How much time (in hours) do you spend on average on COVID-19 information per day? (Including reading outbreak information on mobile or TV news, discussing the progress of the outbreak with family and friends, etc.)**

No time spent (zero time) Less than 30 min 30 min - 1 Hour

1 - 3 hours More than 3 hours

**كم معدّل الوقت (بالساعات) الذي تمضيه يومياً للحصول ​​على معلومات عن كوفيد -19 ؟ (بما في ذلك قراءة أخبار التفشي على الهاتف المحمول أو التلفزيون، ومناقشة تقدم تفشي المرض مع العائلة والأصدقاء، وما إلى ذلك)**

لم يتم إنفاق الوقت (صفر وقت) أقل من 30 دقيقة بين 30 دقيقة – ساعة

1 - 3 ساعات أكثر من 3 ساعات

**SECTION 2: IDEAS AND ATTITUDES TOWARD COVID-19 PATIENTS OR SUSPECTED CASES.**

**تتعلق الأسئلة التالية بالأفكار والمواقف التي لديك بخصوص مرضى COVID-19 أو الحالات المشتبه بها.**

| **Stigma discrimination scale: Please answer the following questions** | | | | | | |
| --- | --- | --- | --- | --- | --- | --- |
|  |  | Strongly agree  أوافق بشدة | Agree  أوافق | Neutral  محايد | Disagree  أعارض | Strongly disagree  أعارض بشدة |
|  | You feel it is not worthwhile for you to serve persons who contracted COVID-19  تشعر أنه ليس من المجدي بالنسبة لك خدمة الأشخاص الذين أصيبوا بـ COVID-19 |  |  |  |  |  |
|  | When a person with COVID-19 asks you for help, you would take the initiative to care for him/her.  عندما يطلب منك شخص مصاب بـ COVID-19 المساعدة ، فأنت تأخذ زمام المبادرة لرعايته. |  |  |  |  |  |
|  | You feel you have the right to refuse to serve people with COVID-19 in order to protect yourself from being infected.  تشعر أن لديك الحق في رفض خدمة الأشخاص المصابين بـ COVID-19 من أجل حماية نفسك من الإصابة. |  |  |  |  |  |
|  | People with current COVID-19 are dangerous to the society.  الأشخاص المصابون بـ COVID-19 يشكلون خطراً على المجتمع. |  |  |  |  |  |
|  | People with current COVID-19 are no different from anybody else.  لا يختلف الأشخاص المصابون بـ COVID-19 عن أي شخص آخر. |  |  |  |  |  |
|  | Nobody deserves to be COVID-19 positive.  لا أحد يستحق أن يكون مصاباً بفيروس COVID-19. |  |  |  |  |  |
|  | People with current COVID-19 should be ashamed of themselves.  يجب أن يخجل الأشخاص المصابون بـ COVID-19 من أنفسهم. |  |  |  |  |  |
|  | People with current COVID-19 have nothing to feel guilty about.  ليس لدى الأشخاص المصابين بـ COVID-19 ما يشعرون بالذنب حياله. |  |  |  |  |  |
|  | Families of people living with COVID-19 should be ashamed.  يجب أن يخجل أفراد عائلة الأشخاص المصابين بـ COVID-19. |  |  |  |  |  |
|  | People with current COVID-19 should not have the same freedoms as other people.  لا ينبغي أن يتمتع الأشخاص المصابون بـ COVID-19 بنفس الحريات مثل الآخرين. |  |  |  |  |  |
|  | Do you relate any locations or ethnicity to the disease such as “Wuhan Virus”, “Chinese Virus” or “Asian Virus”?  هل تربط أي مواقع أو عرق بالمرض مثل "فيروس ووهان" أو "الفيروس الصيني" أو "الفيروس الآسيوي"؟ |  |  |  |  |  |

| **Bullying scale: Please answer the following questions** | | | | | | |
| --- | --- | --- | --- | --- | --- | --- |
|  |  | **Never**  **أبدا** | **Once or twice**  **مرة أو مرتين** | **A few times (between 3 and 5)**  **مرات قليلة (بين 3 و 5 )** | **Several times (between 6 and 10)**  **عدة مرات (بين 6 و 10)** | **Many times (more than 10)**  **مرات عديدة**  **(أكثر من 10 مرات)** |
|  | I teased someone in my surrounding when I knew he tested COVID-19 positive  لقد قمت بإزعاج شخصاً ما في محيطي عندما علمت أنه مصاباً بفيروس COVID-19 |  |  |  |  |  |
|  | I have called someone having COVID-19 with hurtful names  لقد أطلقت على شخص مصاب بفيروس COVID-19 أسماء مؤذية |  |  |  |  |  |
|  | I hurt someone with COVID-19/ or suspected having COVID-19 by trying to break up a friendship  لقد آذيت شخصاً مصاباً أو مشتبه بأنه مصاب بفيروس COVID-19 من خلال محاولة إنهاء صداقة |  |  |  |  |  |
|  | I have ignored someone having COVID-19  لقد تجاهلت شخصاً مصاباً بفيروس COVID-19 |  |  |  |  |  |
|  | I have refused to talk with someone having COVID-19  لقد رفضت التحدث مع شخص مصاب بفيروس COVID-19 |  |  |  |  |  |
|  | I would not let someone having COVID-19 to join my friendship group  لن أسمح لأي شخص مصاب بـ COVID-19 بالانضمام إلى مجموعة الصداقة الخاصة بي |  |  |  |  |  |
|  | I have told lies and/or spread rumors about someone with COVID-19 to make their friends or others not talk to him  لقد قلت أكاذيب و / أو نشرت شائعات عن شخص مصاب بـ COVID-19 لجعل أصدقائه أو الآخرين لا يتحدثون معه |  |  |  |  |  |
|  | I have insulted or ridiculed someone with COVID-19 on social networks or groups like WhatsApp and Facebook to really annoy him  لقد أهنت أو سخرت من شخص مصاب بـ COVID-19 على وسائل التواصل الاجتماعي مثل WhatsApp أو Facebook لإزعاجه |  |  |  |  |  |
|  | I have sent or posted mean or hurtful pictures/videos on social networks to someone with COVID-19  لقد أرسلت أو نشرت صوراً / مقاطع فيديو مسيئة أو مؤذية لشخص مصاب بفيروس COVID-19 على وسائل التواصل الاجتماعي |  |  |  |  |  |
|  | I have ignored someone with COVID-19 and did not answer messages or things he shared in groups or social networks, just to make him feel bad  لقد تجاهلت شخصاً مصاباً بـ COVID-19 ولم أجب على الرسائل أو الأشياء التي شاركها في المجموعات أو عبر وسائل التواصل الاجتماعي، فقط لجعله يشعر بالسوء |  |  |  |  |  |
|  | I have eliminated or blocked someone with COVID-19 from groups to leave him/her without any friends  لقد قمت بإزالة أو حظر شخص مصاب بـ COVID-19 من المجموعات على وسائل التواصل الاجتماعي لأتركه بدون أي أصدقاء |  |  |  |  |  |

**SECTION 3: REACTION AND FEELINGS TOWARD COVID-19**

| **Fear of COVID-19 scale: Please respond to each item and choose the best response that reflects how you feel, think, or act toward COVID-19.**  **يرجى الاجابة على كل سؤال واختيار أفضل اجابة تعكس شعورك أو تفكيرك أو تصرفك تجاه فيروس كورونا COVID-19** | | | | | | |
| --- | --- | --- | --- | --- | --- | --- |
|  | | Strongly disagree  أعارض بشدّة | Disagree  أعارض | Neither agree nor disagree  لا أوافق ولا أعارض | Agree  أوافق | Strongly agree  أوافق بشدّة |
|  | I am most afraid of coronavirus-19.  أنا خائف من فيروس كورونا. |  |  |  |  |  |
|  | It makes me uncomfortable to think about coronavirus-19.  التفكير بفيروس كورونا يجعلني غير مرتاح. |  |  |  |  |  |
|  | My hands become clammy when I think about coronavirus-19.  عند التفكير بفيروس كورونا تتعرّق يديّ عند التفكير بفيروس كورونا. |  |  |  |  |  |
|  | I am afraid of losing my life because of coronavirus-19.  أخاف أن أفقد حياتي بسبب فيروس كورونا |  |  |  |  |  |
|  | When watching news and stories about coronavirus-19 on social media, I become nervous or anxious.  عندما أشاهد الأخبار والروايات المتعلّقة بفيروس كورونا على مواقع التواصل الإجتماعي، أصاب بالعصبيّة والتّوتر. |  |  |  |  |  |
| 6 | I cannot sleep because I’m worrying about getting coronavirus-19.  لا أستطيع النّوم خوفاً من الإصابة بفيروس كورونا |  |  |  |  |  |
| 7 | My heart races or palpitates when I think about getting coronavirus-19.  تتسارع نبضات قلبي و يخفق بشدّة عند التفكير بفيروس كورونا. |  |  |  |  |  |

| **Anxiety scale: How often have you experienced the following feelings over the last 2 weeks?**  **كم مرة عانيت من المشاعر التالية خلال الأسبوعين الماضيين؟** | | | | | | |
| --- | --- | --- | --- | --- | --- | --- |
|  | | Not at all (0)  أبداً (0) | Rare, less than a day or two (1)  نادراً، أقل من يوم أو يومين (1) | Several days (2)  عدة أيام (2) | More than 7 days (3)  أكثر من 7 أيام (3) | Nearly every day  over the last 2 weeks (4)  كل يوم تقريباً  خلال الأسبوعين الماضيين (4) |
| 1 | I felt dizzy, lightheaded, or faint, when I read or listened to news about the coronavirus  شعرت بدوار في الرأس، دوخة أو إغماء عندما قرأت او استمعت إلى أخبار عن فيروس كورونا |  |  |  |  |  |
| 2 | I had trouble falling or staying asleep because I was thinking about the coronavirus  واجهت مشكلة في النوم أو البقاء نائماً لأنني كنت أفكر في فيروس كورونا |  |  |  |  |  |
| 3 | I felt paralyzed or frozen when I thought about or was exposed to information about the coronavirus  شعرت بالشلل أو التجمد عندما فكرت أو قرأت معلومات عن فيروس كورونا |  |  |  |  |  |
| 4 | I lost interest in eating when I thought about or was exposed to information about the coronavirus  فقدت الاهتمام بتناول الطعام عندما فكرت أو قرأت معلومات عن فيروس كورونا |  |  |  |  |  |
| 5 | I felt nauseous or had stomach problems when I thought about or was exposed to information about the coronavirus  شعرت بالغثيان أو عانيت من مشاكل في المعدة عندما فكرت أو قرأت معلومات عن فيروس كورونا |  |  |  |  |  |

**SECTION 4: KNOWLEDGE, ATTITUDE, AND PRACTICE TOWARD COVID-19**

**Knowledge Section:**

1. **Which of the following is true about COVID-19? (check all that applies)**

Person to person transmission can occur by droplets

Transmission can be airborne

Most common signs and symptoms include fever, diarrhea, and dyspnea

I do not know

**أي مما يلي ينطبق على COVID-19؟ (اختر كل ما ينطبق)**

يمكن أن يحدث الانتقال من شخص لآخر بواسطة الرذاذ

يمكن أن تنتقل العدوى في الهواء

تشمل معظم العلامات والأعراض الأكثر شيوعاً الحمى والإسهال وضيق التنفس

لا أعرف

1. **For how long should a person be isolated in case of COVID-19 infection suspicion (mild symptoms or contact with an infected persons)?**

7 days 10 days 14 days 20 days >20 days I do not know

**لكم من الوقت يجب عزل الشخص في حالة الاشتباه في الإصابة بـ COVID-19 (أعراض خفيفة أو مخالطة أشخاص مصابين)؟**

7 أيام 10 أيام 14 يوماً 20 يوماً أكثر من 20 يوماً لا أعرف

1. **Can someone who has been quarantined for COVID-19 spread the illness to others?**

No, if the quarantine period is less than 14 days

No, if the quarantine period is 14 days or more

I do not know

**هل يمكن لشخص تم عزله بسبب COVID-19 أن ينقل العدوى إلى الآخرين؟**

لا، إذا كانت فترة الحجر أقل من 14 يومًا

لا، إذا كانت فترة الحجر الصحي 14 يوماً أو أكثر

لا أعرف

1. **What are the steps to take to protect yourself? (check all that applies)**

Wash your hands with soap and water for at least 10 seconds

Wash your hands with soap and water for at least 20 seconds

Avoid close contact; put distance between yourself and other people (1.5-2 meters)

Wear a facemask and stay home if you have any respiratory symptom

No need to clean and disinfect solid objects (tables, doorknobs, desks, phones, etc.)

I do not know

**ما هي الخطوات التي يجب اتخاذها لحماية نفسك؟ (اختر كل ما ينطبق)**

اغسل يديك بالصابون والماء لمدة 10 ثوانٍ على الأقل

اغسل يديك بالصابون والماء لمدة 20 ثانٍية على الأقل

تجنب الاتصال الوثيق. ضع مسافة بينك وبين الآخرين (1.5-2 متر)

ارتدِ قناعاً للوجه وأبق في المنزل إذا كان لديك أي أعراض تنفسية

لا حاجة لتنظيف وتطهير الأشياء الصلبة (الطاولات، مقابض الأبواب، المكاتب، الهواتف، إلخ.)

لا أعرف

1. **Can a person test negative and later test positive for COVID-19?**

Yes No I do not know

**هل يمكن لفحص الـ COVID-19 أن يصبح إيجابياً بعد أن كان سلبياً في السابق؟**

نعم لا لا أعرف

1. **If a suspected person tests negative but has no symptoms (check all that applies):**

It is definitely a true negative

It can be a false negative in the pre-symptomatic phase

I do not know how to interpret this test result, I refer to a specialist

**إذا كان فحص الـ COVID-19 للشخص المشتبه به سلبياً ولكن ليس لديه أعراض (اختر كل ما ينطبق):**

إنه بالتأكيد سلبي حقيقي

يمكن أن يكون سلبي خاطئ في مرحلة ما قبل الأعراض

لا أعرف كيف أفسر نتيجةهذا الاختبار، فأستشير أخصائي

1. **Is the person at risk if he/she goes to a funeral of someone who died of COVID-19?**

Yes, since he will meet the dead person close contacts

No known risk currently

I do not know

**هل الشخص معرض للخطر إذا ذهب إلى جنازة شخص مات بسبب الCOVID-19؟**

نعم ، حيث أنه سيلتقي بمقربين من الفقيد

لا يوجد خطر معروف حالياً

لا أعرف

1. **When can Confirmed COVID-19 cases be released from quarantine?**

Following one negative PCR test after resolution of symptoms

Following two negative PCR tests 24 hours apart after resolution of symptoms

Following four negative PCRs on three consecutive days after resolution of symptoms

I do not know

**متى يمكن الإفراج عن حالات COVID-19 المؤكدة من الحجر الصحي؟**

بعد اختبار PCR سلبي واحد من بعد انتهاء الأعراض

بعد اختبارين سلبيين للPCR بفارق 24 ساعة من بعد انتهاء الأعراض

بعد أربعة اختبارات سلبية للPCR في ثلاثة أيام متتالية من بعد انتهاء الأعراض

لا أعرف

1. **Do you think you should avoid contact with pets or other animals if you are sick with COVID-19?**

Yes No I do not know

**هل يجب أن تتجنب الاتصال بالحيوانات الأليفة أو الحيوانات الأخرى إذا كنت مصابا بـ COVID-19؟**

نعم لا لا أعرف

1. **Who are the people most vulnerable to COVID-19? (Check all that applies)**

Elderly People with underlying illness and co-morbidities

Children Adolescents Adults

**من هم الأشخاص الأكثر عرضة للإصابة بـ COVID-19؟ (اختر كل ما ينطبق)**

كبار السن الأشخاص الذين يعانون من أمراض مزمنة وأمراض مرافقة

الأطفال المراهقين الراشدين

1. **What are the most common symptoms related to COVID-19?**

Fever, productive cough, rhinorrhea

Fever, dry cough, dyspnea

Fever, diarrhea, pharyngitis

None of the above

I do not know

**ما هي أكثر الأعراض شيوعًا المرتبطة بـ COVID-19؟**

الحمى، السعال المنتج للبلغم وسيلان الأنف

الحمى، السعال الجاف وضيق التنفس

الحمى، الإسهال والتهاب البلعوم

لا شيء مما ذكر بالأعلى

لا أعرف

1. **Which of the diseases below are due to coronavirus? (Check all that applies)**

Middle East respiratory syndrome (MERS)

Severe acute respiratory syndrome coronavirus 2 (SARS-CoV-2)

Influenza A (H1N1)

Severe acute respiratory syndrome (SARS)

I don’t know

**أي من الأمراض المذكورة أدناه ناتجة عن فيروس كورونا؟ (إختر كل ما ينطبق)**

متلازمة الشرق الاوسط التنفسية (MERS-CoV)

الفيروس التاجي المستجد (SARS-CoV-2)

الإنفلونزا (H1N1)

متلازمة الالتهاب التنفسي الحاد (SARS)

لا أعرف

1. **Is coronavirus the same as the common flu?**

Yes No I do not know

**هل فيروس كورونا هو نفسه فيروس الأنفلونزا ؟**

نعم لا لا أعرف

1. **What is the incubation period of COVID-19?**

1-14 days 1-3 months

2-21 days I don't know

**ما هي فترة حضانة فيروس كورونا؟**

1-14 يوماً 1-3 أشهر

2-21 يوماً لا أعرف

1. **Mode of transmission of coronavirus**

Air droplets (from patient sneezing/coughing)

Close contact with people who have the virus

Contact with contaminated surfaces

Mosquitos/flies bites

I don't know

**طريقة انتقال ال COVID-19**

قطرات الهواء (من عطاس / سعال المريض)

الاتصال الوثيق بالأشخاص المصابين بالفيروس

ملامسة الأسطح الملوثة

لدغات البعوض / الذباب

لا أعرف

1. **Is hand washing important?**

Yes No Maybe I do not know

**هل غسل اليدين مهم؟**

نعم لا ربما لا أعرف

1. **For how long should you wash your hands**

5 minutes 1-3 minutes 20 seconds to 1 minute

3-5 minutes Less than 20 seconds I don't know

**لكم من الوقت يجب أن تغسل يديك**

5 دقائق دقيقة إلى 3 دقائق 20 ثانية إلى دقيقة واحدة

3-5 دقائق أقل من 20 ثانية لا أعرف

1. **Can a person infected with coronavirus get infected a second time?**

Yes No Maybe I do not know

**هل يمكن لشخص أصيب بـ COVID-19 أن يصاب مرة ثانية؟**

نعم لا ربما لا أعرف

1. **Can a person recovered from COVID-19 transmit the infection to others?**

Yes No Maybe I do not know

**هل يمكن لأي شخص شفي من COVID-19 أن ينقل العدوى للآخرين؟**

نعم لا ربما لا أعرف

1. **Can you catch the virus from surfaces and tools contaminated with COVID-19?**

Maybe No I do not know

**هل يمكن أن ينتقل إليك الفيروس بعد ملامستك الأسطح والأدوات الملوثة بفيروس كورونا؟**

ربما لا لا أعرف

**Practice Section:**

|  |  | **Never** | **Rarely** | **Neutral** | **Occasionally** | **Always** |
| --- | --- | --- | --- | --- | --- | --- |
|  | In the last few days, have you worn a mask when you were in a crowded place?  في الأيام القليلة الماضية، هل ارتديت كمامة عندما كنت في مكان مزدحم؟ |  |  |  |  |  |
|  | In the last few days, have you implemented physical distancing when you were in the crowd?  في الأيام القليلة الماضية، هل نفذت التباعد الاجتماعي عندما كنت في الحشد؟ |  |  |  |  |  |
|  | In the last few days, have you used hand sanitizer when you were in crowded places?  في الأيام القليلة الماضية، هل استخدمت معقم اليدين عندما كنت في الأماكن المزدحمة؟ |  |  |  |  |  |
|  | In the last few days, have you washed your hands with soap after going to a crowded place?  في الأيام القليلة الماضية، هل غسلت يديك بالصابون بعد الذهاب إلى مكان مزدحم؟ |  |  |  |  |  |
|  | Do you use tissues or cover your mouth during coughing/sneezing?  هل تستخدم المناديل الورقية أو تغطي فمك أثناء السعال / العطس؟ |  |  |  |  |  |
|  | Do you replace the face mask after a single use?  هل تستبدل الكمامة بعد استخدام واحد؟ |  |  |  |  |  |
|  | Do you avoid touching face and eyes?  هل تتجنب ملامسة الوجه والعينين؟ |  |  |  |  |  |
|  | Do you maintain social distance (or home quarantine)?  هل تحافظ على المسافة الاجتماعية (أو الحجر الصحي بالمنزل)؟ |  |  |  |  |  |
|  | Do you eat healthy food focusing on outbreak?  هل تأكل طعاماً صحياً |  |  |  |  |  |
|  | Do you obey all government rules related to the COVID?  هل تلتزم بجميع التوصيات الحكومية المتعلقة بـ COVID؟ |  |  |  |  |  |
|  | Do you Clean/disinfect mobile phone  هل تقوم بتنظيف / تطهير الهاتف المحمول |  |  |  |  |  |
|  | Do you avoid contact with people at risk?  هل تتجنب التواصل بالأشخاص المعرضين للخطر |  |  |  |  |  |
|  | Do you avoid groups?  هل تتجنب المجموعات |  |  |  |  |  |
|  | Do you avoid shaking hands?  هل تتجنب مصافحة الأيدي |  |  |  |  |  |
|  | Do you stay at home when it is requested by the government?  هل تلتزم المنزل عندما يطلب منك ذلك من قبل وزارة الصحة |  |  |  |  |  |
|  | Do you stay at home with symptoms?  هل تبقى في المنزل حين ظهور العوارض |  |  |  |  |  |
|  | If a friend or relative of yours were to have frequent contact with people with COVID-19, you prefer not to meet him/her.  إذا كان صديقك أو قريبك على اتصال متكرر بأشخاص مصابين بـ COVID-19، فأنت تفضل عدم مقابلته/ها. |  |  |  |  |  |
|  | Even if you only had to speak to a person with COVID-19, you would wear a mask to prevent infection.  حتى إذا كان عليك فقط التحدث إلى شخص مصاب بـ COVID-19، فعليك ارتداء قناع لمنع العدوى. |  |  |  |  |  |
|  | For reasons of general safety, you think you should not get near a person with COVID-19.  لأسباب تتعلق بالسلامة العامة، تعتقد أنه لا يجب عليك الاقتراب من شخص مصاب بـ COVID-19. |  |  |  |  |  |
|  | You are not against serving persons with COVID-19, but would try your best not to get too close to them.  أنت لست ضد خدمة الأشخاص المصابين بـCOVID-19، لكنك ستبذل قصارى جهدك حتى لا تقترب منهم كثيراً. |  |  |  |  |  |
|  | People with current COVID-19 should be isolated and cannot actively participate in the social events in this community.  يجب عزل الأشخاص المصابين بـ COVID-19 ولا يمكنهم المشاركة بنشاط في الأماكن الاجتماعية |  |  |  |  |  |
|  | A person with COVID-19 should not be allowed to work with other people.  لا ينبغي السماح لأي شخص مصاب بـ COVID-19 بالعمل مع أشخاص آخرين. |  |  |  |  |  |
|  | Families of people living with COVID-19 should be isolated.  يجب عزل أفراد عائلة الأشخاص المصابين بـ COVID-19. |  |  |  |  |  |
|  | It is reasonable for an employer to exclude/isolate a person with COVID-19.  من المعقول أن يستبعد صاحب العمل / يعزل شخصاً مصاباً بـ COVID-19. |  |  |  |  |  |

**Attitudes Section:**

|  |  | **Disagree** | **Not sure** | **Agree** |
| --- | --- | --- | --- | --- |
|  | Do you think social distancing/self-isolation is an effective measure to reduce the spread of COVID-19?  هل تعتقد أن التباعد الاجتماعي / العزلة الذاتية هو إجراء فعال للحد من انتشار COVID-19؟ |  |  |  |
|  | Do you think that regular hand washing, maintaining social distancing and use of masks can protect you from coronavirus?  هل تعتقد أن غسل اليدين بانتظام والتباعد الاجتماعي واستخدام الكمامات يمكن أن يحميك من فيروس كورونا؟ |  |  |  |
|  | Do you think lockdown will be helpful in controlling the coronavirus disease?  هل تعتقد أن الإغلاق (Lockdown) سيكون مفيداً في السيطرة على مرض فيروس كورونا |  |  |  |
|  | Keeping up with the information regarding the government’s call for COVID-19 preventive efforts is important for the community  تعد مواكبة المعلومات المتعلقة بدعوة الحكومة للجهود الوقائية لـ COVID-19 أمرًا مهمًا للمجتمع |  |  |  |
|  | People with COVID-19 should not be stigmatized in society  لا ينبغي إدانة الأشخاص المصابين بـ COVID-19 في المجتمع |  |  |  |
|  | People with COVID-19 who isolate themselves show that they have a responsibility in preventing the transmission of COVID-19  الأشخاص المصابون بـ COVID-19 الذين يعزلون أنفسهم يظهرون مسؤولية في منع انتقال COVID-19 |  |  |  |

**Additional file 2**

| **Table S1: Sociodemographic characteristics of the study sample** | | |
| --- | --- | --- |
|  | **Frequency** | **Percentage** |
| **Gender** |  |  |
| Male | 82 | 20.2% |
| Female | 323 | 79.8% |
| **Marital status** |  |  |
| Single | 276 | 68.1% |
| Married | 129 | 31.9% |
| **Education level** |  |  |
| School level | 42 | 10.4% |
| University level | 363 | 89.6% |
| **Monthly income** |  |  |
| No income | 185 | 45.7% |
| Low | 53 | 13.1% |
| Intermediate | 75 | 18.5% |
| High | 92 | 22.7% |
| **Employment status** |  |  |
| Employed, Medical field (frontline contact with COVID-19 patients) | 10 | 2.5% |
| Employed, Medical field (non- frontline contact) | 55 | 13.6% |
| Employed, Non-medical | 114 | 28.1% |
| Unemployed | 226 | 55.8% |
| **Family member in the medical field** |  |  |
| Yes | 114 | 28.1% |
| No | 291 | 71.9% |
| **Living place** |  |  |
| Rural | 108 | 26.7% |
| Urban | 297 | 73.3% |
| **Religion** |  |  |
| Christian | 71 | 17.5% |
| Muslim | 254 | 62.7% |
| Druze | 42 | 10.4% |
| Atheist | 1 | 0.2% |
| Refuse to answer | 35 | 8.6% |
| Other | 2 | 0.5% |
| **Diagnosed with COVID-19** |  |  |
| Yes | 41 | 10.1% |
| No | 364 | 89.9% |
| **Tested with COVID-19** |  |  |
| Yes | 164 | 40.5% |
| No | 241 | 59.5% |
| **History of COVID-19 in the family** |  |  |
| Yes | 113 | 27.9% |
| No | 277 | 68.4% |
| I do not know | 15 | 3.7% |
|  | **Mean** | **SD** |
| **Age** | 28.38 | 12.02 |
| **Household crowding index** | 1.14 | .55 |
